# Programmed longevity, youthspan, and juventology

**DOI:** 10.1111/acel.12843

**Published:** 2018-10-17

**Authors:** Valter D. Longo

**Affiliations:** ^1^ University of Southern California Los Angeles California; ^2^ Center for Regenerative Medicine and Stem Cell Research at USC, Keck School of Medicine University of Southern California Los Angeles California; ^3^ IFOM FIRC Institute of Molecular Oncology Milan Italy

**Keywords:** Juventology, gerontology, youthspan, healthspan, Longevity, aging

## Abstract

The identification of conserved genes and pathways that regulate lifespan but also healthspan has resulted in an improved understanding of the link between nutrients, signal transduction proteins, and aging but has also provided evidence for the existence of multiple “longevity programs,” which are selected based on the availability of nutrients. Periodic fasting and other dietary restrictions can promote entry into a long‐lasting longevity program characterized by cellular protection and optimal function but can also activate regenerative processes that lead to rejuvenation, which are independent of the aging rate preceding the restricted period. Thus, a “juventology”‐based strategy can complement the traditional gerontology approach by focusing not on aging but on the longevity program affecting the life history period in which mortality is very low and organisms remain youthful, healthy, and fully functional.

## 1 NUTRITION, GENES, AND HEALTHSPAN

Simple organisms have served as valuable models to understand the fundamental connection between nutrients and the genes that regulate cellular protection and aging. In *E. coli*,* S. cerevisiae,* and *C. elegans*, starvation, the most severe form of dietary restriction, causes a major lifespan extension (Longo & Mattson, 2014). In the yeast *S. cerevisiae*, the effect of the deficiency in amino acids, particularly serine, threonine, and valine but also methionine on Tor‐S6 kinase and other nutrient signaling pathways, explains part of the pro‐longevity effects of starvation (Johnson & Johnson, 2014; Mirisola et al., 2014; Ruckenstuhl et al., 2014). Reduction in glucose levels and the consequent down‐regulation of Ras‐PKA signaling are also key changes mediating the fasting‐dependent effects on yeast aging (Longo & Mattson, 2014). In mammals, nutrient signaling is much more complex than in yeast, but the fundamental role of macronutrients in accelerating aging may be conserved. In fact, deficiency in methionine and other amino acids can reduce growth hormone signaling and consequently the levels of the insulin‐like growth factor 1 (IGF‐1) but also the activity of the mTor‐S6 Kinase pathway analogously to what has been shown in yeast. Not surprisingly, protein or amino acid restriction also extends longevity in flies and mice and recent epidemiological studies indicate that it can reduce the risk of cancer and overall mortality in humans (Grandison, Piper, & Partridge, 2009; Levine et al., 2014; Miller et al., 2005; Song et al., 2016).

These conserved effects of protein/amino acids restriction on longevity are consistent with the reduced morbidity phenotype in both mice and humans with defects in the pathways they activate including growth hormone receptor (GHRD) signaling, whose inactivation causes protection from both cancer and diabetes (Bartke, Sun, & Longo, 2013; Guevara‐Aguirre et al., 2011). Mice with GH or GHR deficiency also display an over 40% longevity extension, which has not yet been determined for human GHRDs (Bartke & Brown‐Borg, 2004). Because mammalian GHRD model organisms are born with this mutation, it is possible that a significant portion of the longevity phenotype is due to developmental effects of the mutation, particularly considering that methionine restriction early in life can result in lifespan extension. However, rapamycin, calorie restriction, or periodic cycles of a Fasting Mimicking Diet (FMD) started at middle age can extend both longevity and healthspan probably at least in part by inhibiting GH‐IGF‐1 and/or mTor‐S6K signaling (Brandhorst et al., 2015; Colman et al., 2009, 2014 ; de Cabo, Carmona‐Gutierrez, Bernier, Hall, & Madeo, 2014; Harrison et al., 2009; Mattison et al., 2017, 2012 ; Miller et al., 2011), strongly suggesting that the involvement of these pro‐growth genes in the acceleration of the aging process can be independent of developmental effects.

## 2 PROGRAMMED LONGEVITY AND JUVENTOLOGY

Many theories have been proposed to explain the aging process ranging from the free radical theory of aging, to the disposable soma, and antagonistic pleiotropy theories. These were formulated to explain why organisms age and are consistent with the acceleration of damage and dysfunction as the force of natural selection declines. However, we can also consider aging to be the result of the end or at least of a partial inactivation of a “longevity program” whose scope is to maintain the organism young (Longo, Mitteldorf, & Skulachev, 2005). This is distinct from the more controversial “programmed aging” theory, in which the aging process has been selected to provide both genetic variability and the nutritional resources to promote fitness (Fabrizio et al, 2004; Herker et al, 2004). Although the existence a longevity program is very much consistent with the natural selection theory and may appear to be just another way to explain aging, it is not because it relates less to senescence and much more to a series of protection, repair, and replacement events aimed at keeping the organism young. I propose that this field can be termed “juventology” (the study of youth) from the Latin iuventus or “the age of youth.”

For example, we know that *S. cerevisiae* grown in glucose medium can survive for ~6 days in a relatively low protection mode. However, when it is switched to water, stress resistance can increase several folds as does lifespan but also the period in which cells are able to reproduce and form colonies (Longo et al., 2005). Thus, there are clearly at least 2 longevity programs that can be selected by yeast cells and which are entered based on the type and level of nutrients in the medium. This is a fundamental distinction from the “aging‐centered” view for two reasons: (a) A longevity program based on the understanding of juventology, such as the alternative lifespan programs entered in response to fasting, may be independent or partially independent of aging. For example, the use of drugs and periodic fasting, both of which target the mTor‐S6K and PKA pathways, can promote regeneration and rejuvenation. Thus, an organism could be aging at a higher rate and yet have a longer healthspan and lifespan by periodically activating regenerative and rejuvenating processes and (b) by shifting the focus from “old or older age” in which dysfunction generates high morbidity and mortality, to the period during which both morbidity and mortality are very low and difficult to detect. For example, human diseases are rare before age 40, but very common after age 65, yet no specific field of science is focusing on how evolution resulted in a program that is so effective for the first 40 years of life and how that program may be extended by dietary, pharmacological, or other interventions. Although developmental biology can include this important field, its focus is on the embryo to adult stage. Thus, juventology complements both gerontology and developmental biology by focusing on the life history period when the force of natural selection is high and function is maximized. This programmed longevity and juventology view can also be helpful in identifying interventions that can extend lifespan without promoting adverse effects. For example, a significant portion of aging research is focused on the effect of free radicals and other oxygen species on macromolecular damage and on the search of antioxidant enzymes and drugs that can counter these effects. Although there is no doubt that free radicals are among the most potent pro‐aging molecules, treatments with rapamycin, spermidine, metformin, and NAD‐related molecules or dietary restrictions have been proven to be the most effective in healthspan extension. Instead of simply targeting specific toxic molecules which can also have beneficial effects such as killing bacteria or cancer cells, the most successful pro‐longevity interventions force the organism into alternate survival phases characterized by the regulation of hundreds of proteins. This is not surprising since they also affect free radical damage by activating existing and highly coordinated programs that increase cellular protection, repair, and regeneration evolved to respond to starvation conditions or other insults (Brandhorst et al., 2015; Cheng et al., 2014; Colman et al., 2009, 2014; Harrison et al., 2009; Longo & Mattson, 2014; Madeo, Eisenberg, Pietrocola, & Kroemer, 2018; Mattison et al., 2017; Miller et al., 2011). In particular, either intracellular regeneration such as that involving autophagy or stem cell‐based regeneration could return the organism to a more youthful state independently of the previous rate or level of oxidative damage.

## 3 JUVENTOLOGY IN RESEARCH AND MEDICINE

**Figure 1:**
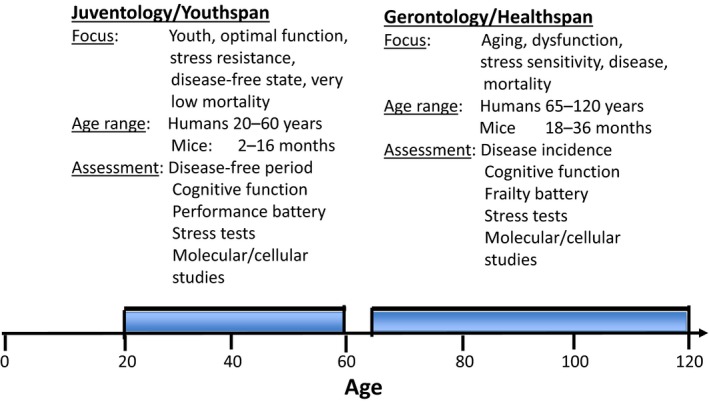
A comparison between the age ranges, focus, and assessments related to juventology and youthspan versus gerontology and healthspan

The juventology strategy could eventually play a central role in medicine by complementing the targeting of specific enzymes or processes with the targeting of the type of programs that allow organisms to remain highly functional and able to reproduce. This program allows yeast to live for 6 days but remain highly protected for 3 days, and mice to live 2.5 years while remaining highly protected and functional for about 1 year . Thus, more research effort could be devoted to extending the human youthspan period. Although a major extension of youthspan may sound unrealistic, it has already been achieved in mice, monkeys and possibly humans treated with dietary restrictions, periodic fasting, pro‐longevity drugs, or bearing specific mutations. However, because most studies focus on lifespan and healthspan, we have very limited information on the effect of these interventions on youthspan. In simple model organisms, in which large populations can be studied, youthspan could be easily determined based on age‐specific mortality but also on the measurement of macromolecular damage, stress resistance, cognitive, and reproductive function. In mice and other mammals, youthspan could be also measured by monitoring stress resistance, mortality, fertility, and macromolecular damage, but these measurements could be complemented by a new set of tests such as running speed and other physical tests (Figure 1). These could partially overlap with tests used to asses frailty in the elderly, but would have to be largely modified to assess whether the function is still above the threshold expected in a young or relatively young person. For example, walking speed could be replaced by running speed and grip strength by a battery of strength tests assessing different muscle groups (Figure 1).

Important directions in this field include in‐depth studies of some of the most remarkable examples of alternate longevity programs leading to dramatic increases in lifespan such as the spore and dauer phases in starved yeast and worms, respectively, or that entered by the very long‐lived queen bees. In higher eukaryotes, it will also be of great interest to understand how hibernation and the exit from hibernation affect youthspan, longevity, and biological age. Clinical applications of this “youth”‐centered view could be focused on the identification of drugs or dietary interventions that maximize not lifespan or even healthspan, but the period of life when damage and even loss of function is difficult to detect. For example, in humans the period in which health is maintained or “healthspan” can be 70 years or higher, but “youthspan” or the period in which the individual's function is maximized and diseases are rare may only reach 40–50 years (Figure 1). To achieve this goal, it will be important to clearly establish the level of molecular damage and dysfunction that distinguishes the period in which an individual remains relatively healthy from the period in which it remains young and fully functional.

## 4 CONCLUSION

Periodic fasting and calorie restriction, by causing a decrease in both proteins/amino acids and glucose levels, promote entry into a stress resistance state which is characterized by the activation of cell protection, regeneration, and rejuvenation processes. Both the protective and regenerative mechanisms are activated in part by the down‐regulation of GH, IGF‐1, mTor‐S6K, and PKA signaling, leading to healthspan extension. Because these states have evolved to withstand periods of starvation, they can be viewed as alternative longevity programs activated to achieve extended “youthspan.” Because their ability to affect youthspan and healthspan can depend on periodic cellular regeneration and autophagy in addition to chronic protection and repair, a juventology‐based approach can complement the classic gerontology approach to focus on the earlier highly functional period while also studying the later progressively dysfunctional life history periods.

## CONFLICT OF INTEREST

Valter Longo has no conflict of interest related to this Commentary.
